# Study of the ichthyotoxic microalga *Heterosigma akashiwo* by transcriptional activation of sublethal marker Hsp70b in Transwell co-culture assays

**DOI:** 10.1371/journal.pone.0201438

**Published:** 2018-08-02

**Authors:** Allisson Astuya, Alejandra Rivera, Karina Vega-Drake, Carla Aburto, Fernando Cruzat, Viviana Ulloa, Teresa Caprile, Juan J. Gallardo-Rodríguez

**Affiliations:** 1 Laboratory of Cell Culture and Marine Genomics, Department of Oceanography, Faculty of Natural and Oceanographic Sciences, University of Concepción, Concepción, Chile; 2 COPAS Sur-Austral Program, Department of Oceanography, Faculty of Natural and Oceanographic Sciences, University of Concepción, Concepción, Chile; 3 Laboratory of Proteomics and Genomics of Marine Organisms, Department of Oceanography, Faculty of Natural and Oceanographic Sciences, University of Concepción, Chile; 4 Laboratory of Axon Guidance, Department de Cell Biology, Faculty of Biology, University of Concepción, Concepción, Chile; 5 Department of Chemical Engineering, Faculty of Engineering, University of Concepción, Concepción, Chile; University of Crete, GREECE

## Abstract

Despite the advance of knowledge about the factors and potential mechanisms triggering the ichthyotoxicity in microalgae, these remain unclear or are controversial for several species (e.g. *Heterosigma*). Neither typical toxicity tests carried out with cell extracts nor direct exposure to harmful species were proved suitable to unravel the mechanism of harm. Ichthyotoxic species show a complex harmful effect on fish, which is mediated through various mechanisms depending on the species. In this work, we present a method to study sub-lethal effects triggered by reactive oxygen species of a population of harmful algae *in vivo* over a fish cell line. To that end, Transwell co-cultures in which causative and target species are separated by a 0.4 μm pore membrane were carried out. This allowed the evaluation of the effect of the released molecules by cells in a rapid and compact test. In our method, the harmful effect was sensed through the transcriptional activation of sub-lethal marker Hsp70b in the CHSE214 salmon cell line. The method was tested with the raphidophyte *Heterosigma akashiwo* and *Dunaliella tertiolecta* (as negative control). It was shown that superoxide intracellular content and its release are not linked in these species. The methodology allowed proving that reactive oxygen species produced by *H*. *akashiwo* are able to induce the transcriptional activation of sub-lethal marker Hsp70b. However, neither loss of viability nor apoptosis was observed in CHSE214 salmon cell line except when exposed to direct contact with the raphidophyte cells (or their extract). Consequently, ROS was not concluded to be the main cause of ichthyotoxicity in *H*. *akashiwo*.

## Introduction

Raphidophyceae include unicellular algae related to considerable mortalities in wild and cultured fish populations, causing substantial losses for the aquaculture industry worldwide [1,2]. Some of the main ichthyotoxic species of this class are *Fibrocapsa japonica*, *Chattonella* spp., *Pseudochattonella* and *Heterosigma akashiwo* [3]. Particularly, *H*. *akashiwo* is one of the harmful algal species that impacts in Japan [4], but it has also been detected in many other countries [5–7].

The toxicological mechanisms responsible for the ichthyotoxic properties of *H*. *akashiwo* are currently under debate [8]. For raphidophytes, the following three main mechanisms have been proposed: i) production of neurotoxins (e.g. brevetoxins) [9–11], ii) high free fatty acids content [12], and iii) production of reactive oxygen species (ROS) [13,14]. ROS such as superoxide (^•^O_2_^-^) and hydrogen peroxide (H_2_O_2_) are constitutively generated by microalgae [13,15], although important differences between species and growth phases have been shown [16,17]. ROS generated during harmful algal blooms have been linked to gill tissue injuries in fish, including epithelial lifting, cell necrosis, and the alteration of chloride cells [14]. These injuries, in turn, produce massive mucus secretion from the gills and physiological responses such as hypoxia and subsequent asphyxia [14]. However, the current understanding is that the observed effects are attributable to a synergistic relation between ROS, polyunsaturated fatty acids (PUFA) and biotoxins [12], and that the causes of ichthytoxicity are strongly species-dependent [8]. So far, most of the tests carried out were based in direct contact of cells, or the extracts thereof, with cell lines [1,12,18–20] or fish [12,21]. Dorantes-Aranda et al. in a pioneering study used a Transwell plate to expose gill cells to living ichthyotoxic microalgae [22]. In their assay, they intended to mimic a fish gill, so gill cells and microalgae were able to have physical contact. Then, released (allelopathic, ROS, etc) molecules and those constitutive of the cell membrane were evaluated together.

Despite of providing useful information, these tests arise two important concerns: i) direct contact hinders the effect of diffusible released molecules and, ii) in the case of using cell extracts, the effect on the target cell or biomarker cannot be extrapolated to toxicity of living cells in their environment unless massive microalgae lysis occurs. It is our belief that toxicity tests where living causative cells could be assayed separately are able to provide a different perspective of ichthyotoxic mechanisms. Heat-Shock proteins (Hsp) are relevant to understand cellular stress response in fish [23]. In particular, the Hsp70b has not only a role in the response to heat shock, but also to other stressors such as heavy metals [24] or ROS [25].

Consequently, the aim of this work was to propose an *in vitro* assay to elucidate the toxic mechanisms of living ichthyotoxic microalgae. Transwell plates were used to allow co-culturing microalgae and fish cell without the possibility of physical contact. The short time of exposure in the assays, permits that only the released biomolecules can be sensed by the selected fish cell line. The potential of this method was tested with the ichthyotoxic microalga *Heterosigma akashiwo*.

## Materials and methods

### Cell cultures and maintenance

Strains CCMP302 (New Zealand) and CCMP2425 (Spain) of *H*. *akashiwo* were used for this study. The ichthyotoxic microalga was obtained from the Provasoli-Guillard National Center for Culture of Marine Phytoplankton, Maine, USA (NCMA). The green microalga *Dunaliella tertiolecta* (strain UTEX999) was obtained from the Culture Collection of Algae, Austin, Texas, USA (UTEX) and was used as a nontoxic control. These strains were supplied from the Chilean Microalgae Culture Collection COPAS SUR Austral (CCM-UdeC) and were stored and maintained at the center FICOLAB (University of Concepción).

All strains were grown in 100 mL Erlenmeyer flasks containing 50 mL of L1 medium (Guillard and Hargraves, 1993). Culture medium were prepared with natural seawater from Chilean coast (35°S) with a salinity of approximately 33.5 PSU. The cultures were initiated at 1,000 cells mL^-1^ of exponentially growing cells previously acclimated to experimental conditions for 10 days. Cultures were maintained in a culture chamber at 15 ± 2°C, with 50 μmol·photon ·m^-2^·s^-1^ in a 16:8 (Light:Dark) photoperiod, and without aeration. Cultures were shaken manually once a day. Cell density was assessed by cell counting in 1 mL Utermöhl chambers. *H*. *akashiwo* extracts were prepared as described in Astuya et al. [11].

CHSE-214 cell line (Chinook salmon embryo) from *Oncorhynchus tshawytscha* embryo was obtained from the European Collection of Cell Cultures (ECACC). This cell line has been previously used in other ichthyotoxicity studies (e.g. [26]). The cells were maintained in Minimal Essential Medium (MEM; Gibco) supplemented with 10% fetal bovine serum (FBS; Invitrogen), 2 mM glutamine (Invitrogen), 100 U·mL^-1^ penicillin (Invitrogen), 100 μg·mL^-1^ streptomycin (Invitrogen), 2.5 μg·mL^-1^ fungizone (Invitrogen). Cultures were maintained at 20°C without CO_2_ injection.

### ROS determination

#### Intracellular ROS determination using a fluorescent probe

Relative levels of ROS were measured using 2´,7´dichlorodihydrofluorescein diacetate (H_2_DCFDA; Invitrogen), which in the presence of ROS is transformed into the highly fluorescent compound 2´,7´dichlorofluorescein (DCF). This probe has been previously used to study causes of ROS in raphidophytes [1] and dinoflagellates [27]. The assay was performed according to a previously described method [28] with the following modifications: microalga cells in the exponential (7 days of culturing) and stationary (14 days of culturing) phases were inoculated in 96-well microplates at different cell densities (1,000–15,000 cells·mL^-1^) with 10 mM of H_2_DCFDA in L1 medium. Control wells were inoculated with just microalga cells or the H_2_DCFDA solution. Emitted fluorescence was measured over a 6 h time-course assay using a FLx800 Multi-Detection Microplate Reader (BioTek Instruments, Inc.) with excitation and emission filters of 485/20 and 528/20 nm, respectively. DCF fluorescence data was expressed as absolute fluorescent units, and background fluorescence of t = 0 was subtracted from each determination. Additionally, cellular fluorescence was photographed with a laser confocal Nikon Eclipse TE2000-U microscope.

#### Quantitative intracellular superoxide determination

Quantitative determination of intracellular superoxide was performed using the nitroblue tetrazolium (NBT) reduction assay as previously described [29], but with slight modifications. NBT is a cell permeable substrate that is reduced by intracellular superoxide, thus generating an insoluble blue-colored formazan precipitate, which can be dissolved and quantified. Briefly, 5·10^5^ cells were pooled and incubated with 1 mg·mL^-1^ NBT in L1 medium and incubated at 18°C for 1, 2, and 3 h. Cells were then centrifugated at 1,000 rpm for 1 min, washed with L1 medium, and centrifuged to obtain hard pellet at 5,000 rpm for 1 min. Unreduced NBT was eliminated through dehydration by applying 70% methanol for 15 min followed by air drying. Dry pellets were then treated with KOH (2 M) and dimethyl sulfoxide to dissolve formazan aggregates. Data quantification was obtained by measuring absorbance at 630 nm (FLx800 Microplate Reader, BioTek Instruments, Inc.) and using a calibration curve (1–10 nmol NBT). Controls included cells without NBT treatment and nontoxic microalga.

#### Quantitative extracellular superoxide determination

Extracellular superoxide was quantified by monitoring the reduction of cytochrome C. Cell suspensions (0.05, 0.1, 0.25, and 0.5·10^6^ cells) in 1 mL of L1 culture medium and containing cytochrome C at a concentration of 0.5 mg·mL^-1^ were incubated for 6 h at 18°C. Cells were then centrifuged at 1,000 g, and 200 μL of the supernatants were used to measure cytochrome C reduction in a 96-well microplate reader (FLx800, BioTek Instruments, Inc) by determining the change in absorbance at 550 nm. An extinction coefficient of 21,000 M^-1^·cm^-1^ was used. The absorbance values from determinations without cells, corresponding to each time point used, were subtracted from the data. Controls included the omission of cytochrome C and the nontoxic microalga mentioned above.

### Measurements in CHSE-214 cells with Transwell co-culture

#### Experimental design

CHSE-214 cells were seeded at a density of 1·10^6^ cells per well in sterile 6-well culture plates 24 h before the assays. The ichthyotoxic microalga (*H*. *akashiwo*) was co-cultured with the CHSE-214 cell line by using a Transwell culture system with 0.4 μm pore polyester membrane inserts (Corning HTS Transwell). The inserts were placed on each well, where the microalgae (1·10^4^ cells·mL^-1^), at either the exponential or stationary phase, were added to a final volume of 3 mL (1.5 mL of MEM and 1.5 mL of L1) and incubated for 6 hours. As a positive control, 0.1 and 1 μM H_2_O_2_ was used. Negative controls were the medium MEM: L1 without microalgae. In addition, we also assayed the effect of the cells without Transwell, supernatant and microalgae extract. Cultures of *H*. *akashiwo* were harvested through filtration using 0.25 mm GF/F filters (Millipore). Supernatants were immediately put in ice and used within 10 to 15 minutes. Also, the cell line was exposed to the supernatant in the presence antioxidant ascorbic acid (50uM). The filters were sonicated during 10 min at 47 kHz (Branson 1210 Ultrasonic Cleaner, USA) in absolute methanol. The crude microalga methanolic extracts were first evaporated until dryness under N_2_ flux at 40ºC (TurboVap Concentration System, Caliper, Hopkinton, USA) and then dissolved in MEM:L1. Experiments were performed four times and determinations were performed in triplicate. After the exposure to microalga extracts CHSE-214 cells were subjected to cell health evaluation with MTT (3-(4,5- dimethylthiazol-2-yl)-2,5-diphenyltetrazolium bromide), percentage of apoptosis with acridine orange staining, also the good condition of the microalgae was observed with microscopy, ensuring morphogenic integrity and swimming movements. Finally, CHSE-214 cells were used to quantitative RT-PCR assays.

#### Quantitative RT-PCR assays

The mRNA levels of the endogenous genes Hsp70b (sub-lethal marker), Catalase, superoxide dismutase (both oxidation markers) and β-actin (housekeeping gene) in CHSE-214 cells were determined by quantitative real-time PCR (qPCR). Total RNA from CHSE-214 cells was prepared using the TRIzol Reagent (Invitrogen) following the manufacturer's instructions. Then, RNA was treated with DNase I (Thermo Scientific Waltham, MA, USA) and used for the cDNA synthesis reaction with the Revertaid *H Minus* First Strand cDNA Synthesis Kit. (Thermo Scientific) according to the manufacturer's instructions. The qPCR assays were carried out using the using the Maxima SYBR Green qPCR Master Mix (Thermo Scientific) in an Eco Real-Time PCR System (Illumina San Diego, CA, USA). The primers used in qPCR reactions were Hsp70b (Heat shock protein 70b; NM_001124745) (forward: 5’-AGGCCCAACCATTGAAGAGA-3’; reverse: 5’-GCAATGTCCAGCAATGCAATA-3’); Catalase (XM_024379116) (forward: 5’ -GGTTCAGACCCTACTCAACAA- 3’; reverse: 5’–GGTGGAAGTTAAGGCATCAC- 3’); Superoxide dismutase (XM_024398220.1; Superoxide dismutase [Cu-Zn]-like or Superoxide dismutase I) (forward: 5’–CCGTATTCTTTGAGCAGGAG- 3’; reverse: 5’–AGCCGTTGGTGTTGTCTC- 3’) and β-actin (forward:5’- CTGGACTTTGAGCAGGAGAT -3’; reverse: 5’- GGAGTTGTAGGTGGTCTCG -3’). The relative expressions of the analysed genes were determined with the comparative ΔΔCt method [30] and normalized with β*-actin*.

### Statistical analysis

All experimental data are presented as means ± SD of at least three independent experiments performed with no less than triplicate determinations. When possible, the paired Student's t-test was used to assess significances between experimental data using Prism 4.0 software (GraphPad Inc.). p<0.05 (*) and p<0.01 (**) were considered significant and highly significant, respectively.

## Results

Growth curves of *Heterosigma* were followed to establish the different growth phases. The cells grew exponentially between days 1 and 10 (S1 Fig). Then, the strain showed long stationary phase, and cell density started to decline from day 40. In this study, 7-day and 14-day old cultures were used for exponential and stationary phase cells, respectively.

### Evaluation of the toxic potential of *H*. *akashiwo* diffusible factors on the salmon cell line CHSE-214 through in vitro Transwell co-incubation assays

CHSE-214 cells exhibited an epithelial cell phenotype with polygonal morphology. Immunocytochemical analysis showed a strong signal for the intermediate filament protein vimentin (S2 Fig), which supported the observation of morphological changes during exposure to the ichthyotoxic microalga. In order to assess if released ROS could elicit detectable cellular damage to fish cells, the effect of exposure to 0.1 μM of H_2_O_2_ on the expression of Hsp70b mRNA in CHSE-214 cells was evaluated at different time points. The results from the time-course assays showed increasing levels of Hsp70b mRNA expression after 1 h, and a plateau was reached between 2–6 h after hydrogen peroxide exposure. A seven-fold increase in mRNA levels was finally observed, as assessed by quantification relative to β-actin expression (S3 Fig).

**Fig 1:**
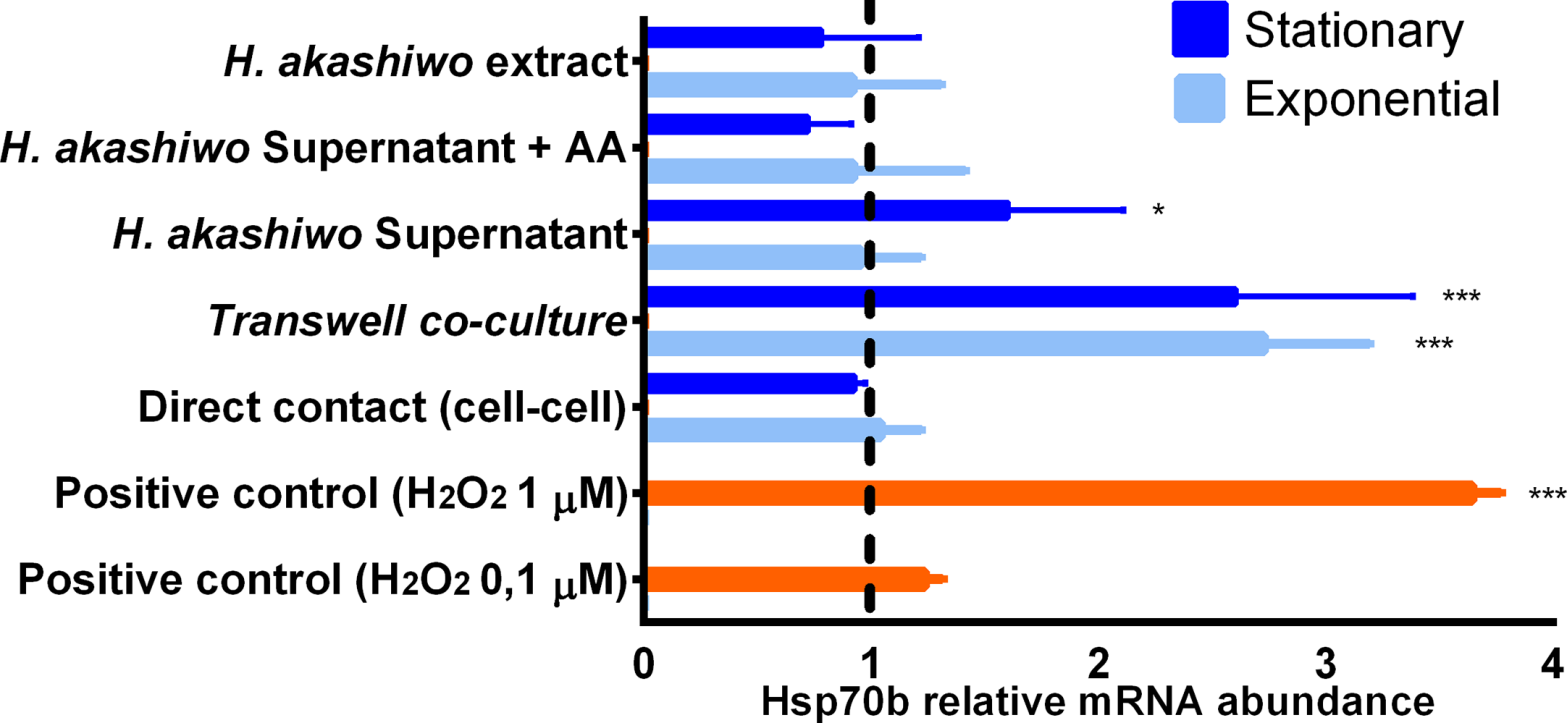
Relative Hsp70b mRNA expression in CHSE-214 cells exposed to *H*. *akashiwo* (CCMP302) (10,000 cell/mL) for 6 h in the exponential (A) and stationary (B) growth phases. Data were relativized to Transwell exposure to *D*. *tertiolecta*.

The effect that soluble compounds released by stationary and exponential *H*. *akashiwo* had on CHSE-214 cells was analyzed using an *in vitro* Transwell co-incubation system. qPCR assays showed an increase in the expression of Hsp70b when the fish cells were co-incubated with *H*. *akashiwo* using the Transwell. H_2_O_2_ treated cells were used as positive control showing concentration dependent response (Fig 1). The ichthyotoxic strain (*H*. *akashiwo* CCMP302) produced a significative increase (p<0.01) when data were relativized to negative control (*D*. *tertiolecta*). There were not significant differences among growth phases. Additionally, CHSE-214 cells were exposed to microalga without Transwell, supernatants and microalga extract. Significative effect in Hsp70b expression was only measured for exponential supernatants, although relative abundance was lower than for the Transwell assay (Fig 1). In this assay, ROS-scavenger ascorbic acid (AA) was used to prove that the expression of Hsp70b mRNA in CHSE-214 cells induced by *H*. *akashiwo* supernatant was due to ROS.

Viability of CHSE-214 cells remained over 90% for more than 6 h for all the treatments (S4 Fig). After 6h, a viability decrease was observed in all samples, including control cultures. However, when we analyzed the acridine orange-stained cells prepared for the apoptosis test, we observed that the cell populations that were directly exposed to the microalga (living cells or total cell extract) showed a considerable increase in their morphological apoptotic-associated characteristics (e.g. condensed chromatin) in comparison to the other assays (Transwell co-culture, supernatant or the H_2_O_2_ controls) (S5 Fig). Similar conclusions can be obtained from the Hoechst staining which revealed nuclear fragmentation (S6 Fig).

**Fig 2:**
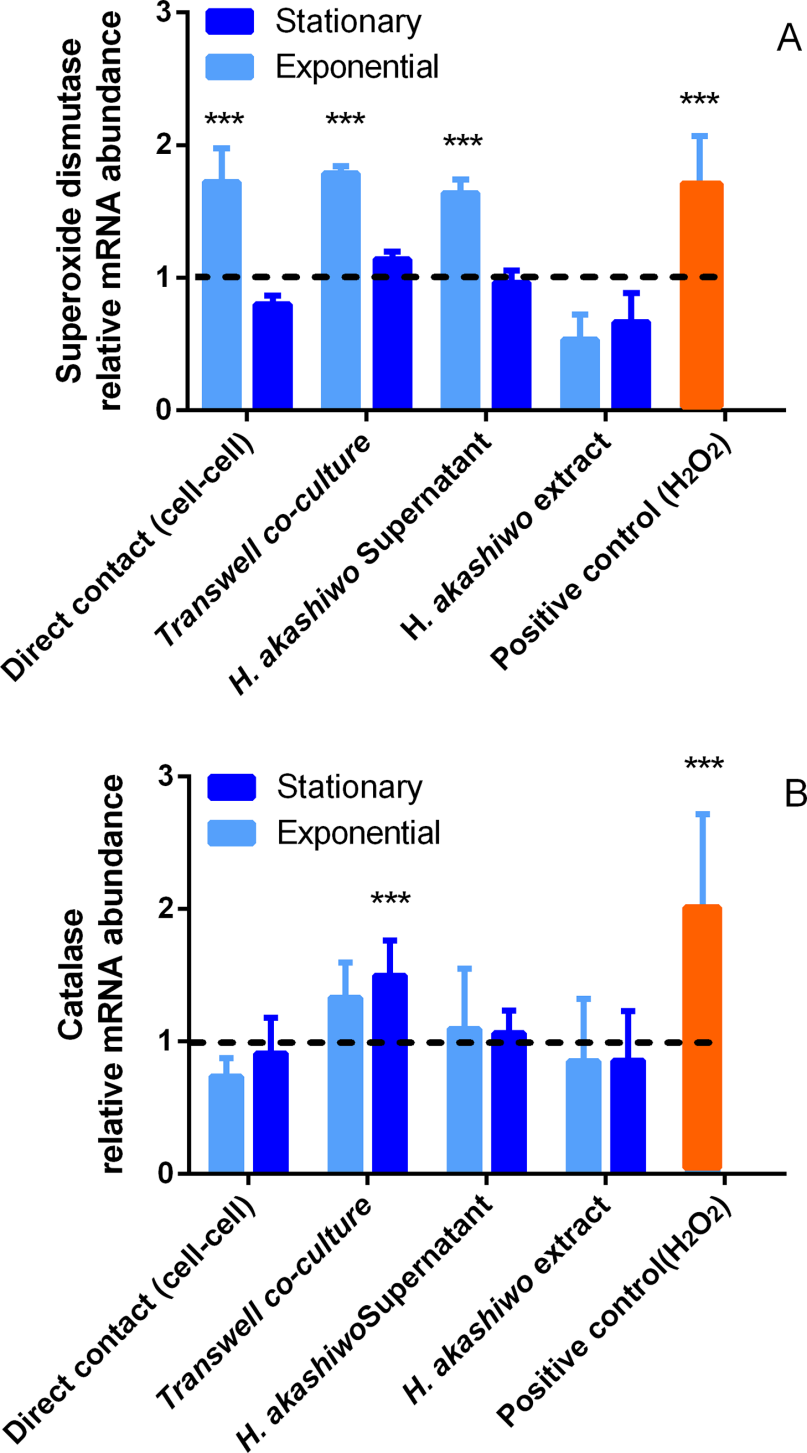
Effect in the expression of SOD and Catalase in CHSE-214 cells exposed to *H*. *akashiwo* microalga (CCMP302).

Since transcriptional activation of Hsp70b could be produced by different triggers [25,31], ROS scavengers production (SOD and Catalase) were evaluated. In Fig 2 values for these enzymes are compared with control cultures and co-cultures. In the case of catalase, there was an increment only in Transwell co-culture with *H*. *akashiwo* (no differences between growth phases). On the other hand, for SOD, besides Transwell co-culture, direct exposure and supernatant of exponential *H*. *akashiwo* had similar effect on the overproduction of this ROS scavenger. Additionally, the total cell extract provoked a significant SOD activity decrease.

### Evaluation of different strains of *H*. *akashiwo* through in vitro Transwell co-incubation with the salmon cell line CHSE-214

**Fig 3:**
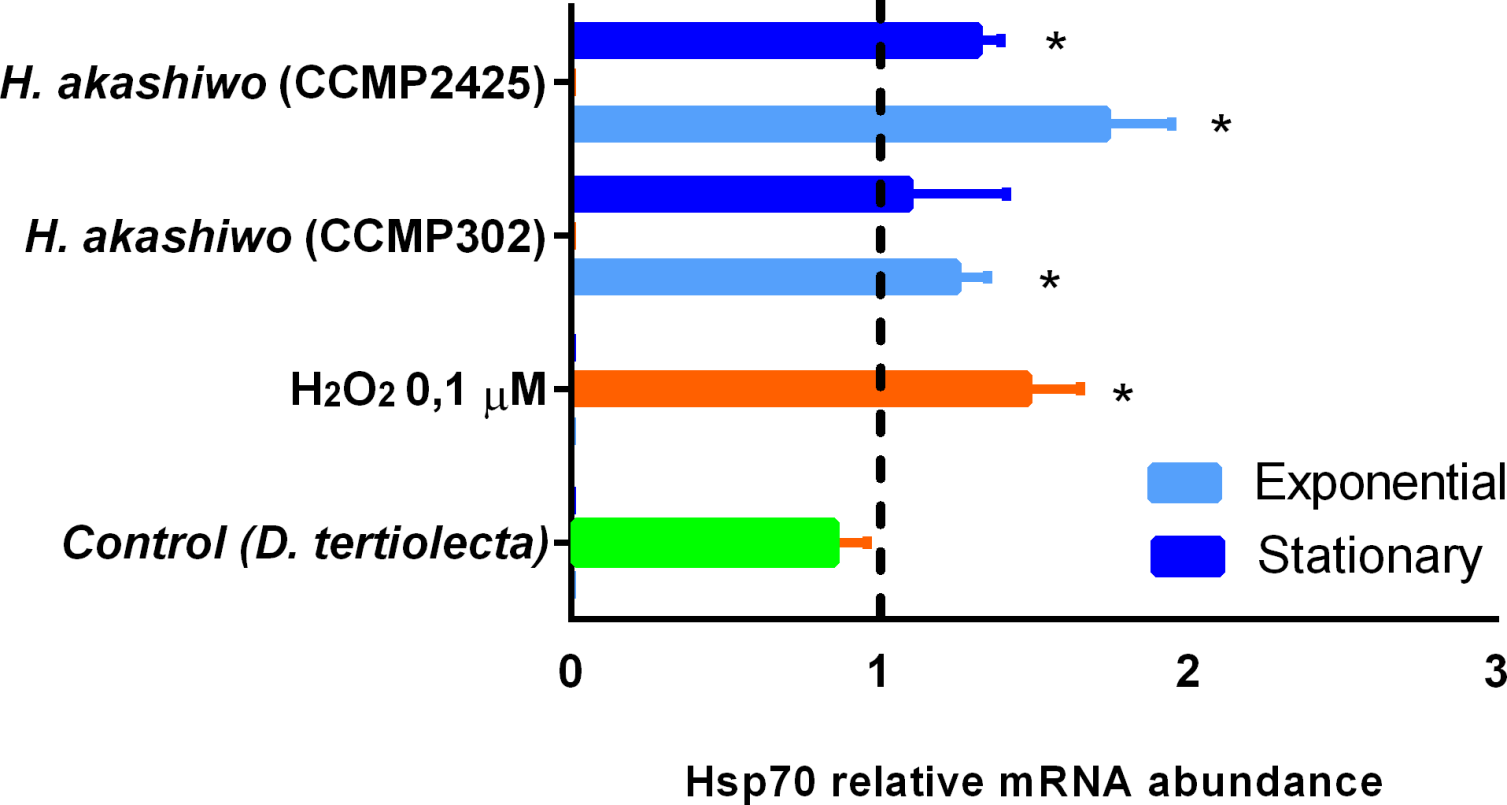
Relative Hsp70b mRNA expression in CHSE-214 cells in *Transwell* co-culture with to *H*. *akashiwo* (CCMP302 and CCMP2425) (10,000 cell/mL) for 30 min in the exponential and stationary growth phases. Data were relativized to unexposed CHSE-214 cells.

The effect that soluble compounds released by different strains of *H*. *akashiwo* had on CHSE-214 cells was analyzed using the *in vitro* Transwell co-incubation system. qPCR assays showed an increase in the expression of Hsp70b when the fish cells were co-incubated with *H*. *akashiwo* using the Transwell. H_2_O_2_ treated cells were used as positive control showing concentration dependent response. Short times (30 min) were enough to show an increase in the expression of Hsp70b when the fish cells were co-incubated with *H*. *akashiwo* (Fig 3). The ichthyotoxic strains (CCMP302 and CCMP2425) produced a significative increase compared to the negative control (*D*. *tertiolecta*). There were differences among growth phases and strains. In both strains, exponential cells had a greater impact on Hsp70 than stationary cells. Strain CCMP2425 showed greater mRNA levels that CCMP302 in both growth phases.

### Quantitative analyses of intracellular and extracellular ROS of *H*. *akashiwo* strains

**Fig 4:**
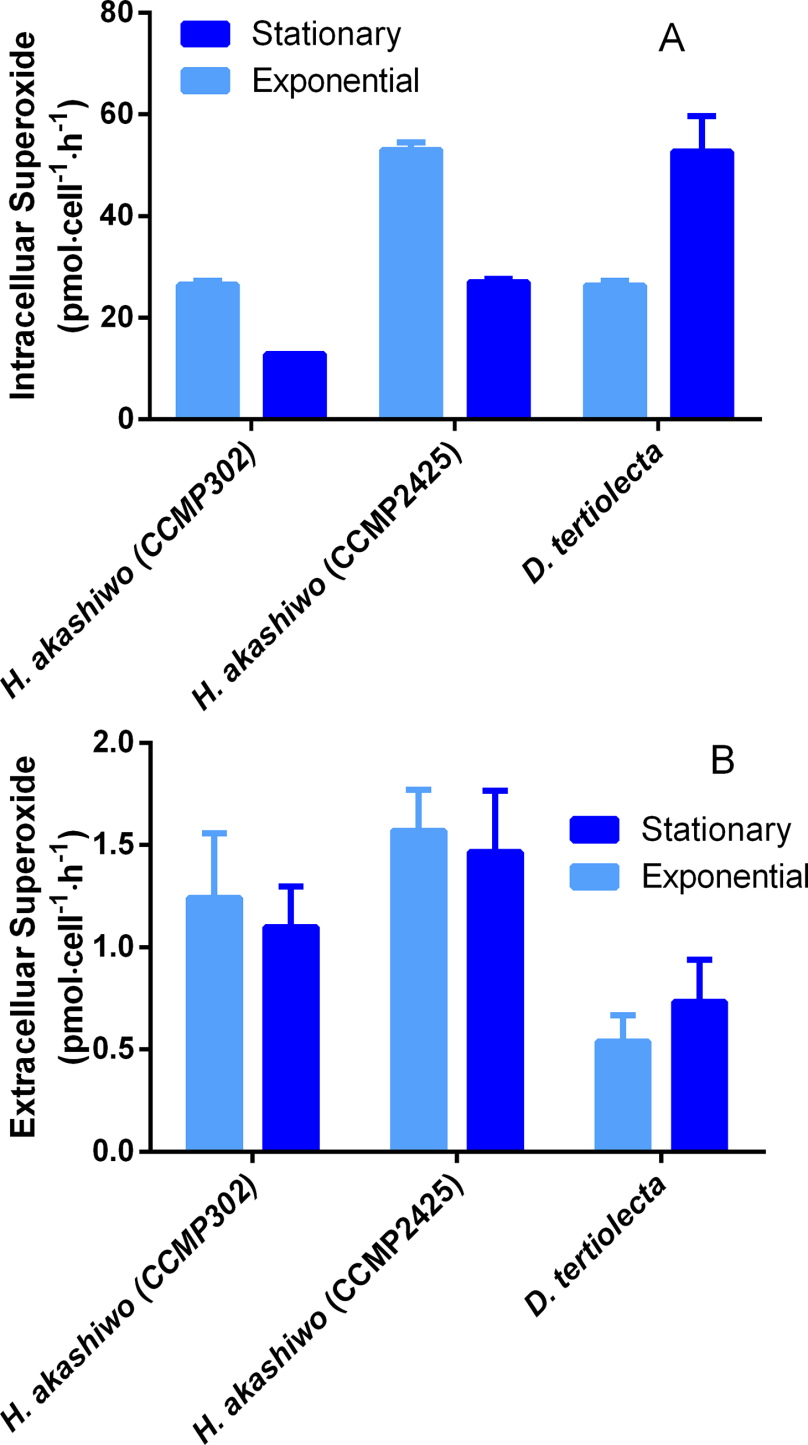
Quantitative determinations of intracellular (A) and extracellular (B) superoxide in *H*. *akashiwo* (CCMP302 and CCMP2425) and *D*. *tertiolecta* as a non-ichthyotoxic control.

A quantitative NBT assay was used for intracellular ROS (superoxide) analysis with *H*. *akashiwo* cells. *D*. *tertiolecta* was used as a non-ichthyotoxic control. Three-hour time course assays showed higher superoxide levels in the exponential growth phases of *H*. *akashiwo* strain compared to stationary cells (Fig 4A). On the contrary, *D*. *tertiolecta* showed higher intracellular superoxide during the stationary phase (Fig 4A). Concentrations of intracellular superoxide were similar between *D*. *tertiolecta* and *H*. *akashiwo* CCMP2425 (although with an opposite pattern between exponential and stationary phases). *H*. *akashiwo* CCMP302 showed much less superoxide per cell than CCMP2425. In order to establish a correlation between intracellular superoxide generation and extracellular-released superoxide, extracellular superoxide was analyzed using non-permeable cytochrome C in experiments with increasing quantities of microalgae. In Fig 4B it can be observed that the two *Heterosigma* strains released extracellular superoxide in higher quantities than *D*. *tertiolecta*. Superoxide release between growth phases showed no significant differences.

### Analysis of ROS production with DCF of *H*. *akashiwo* strains

**Fig 5:**
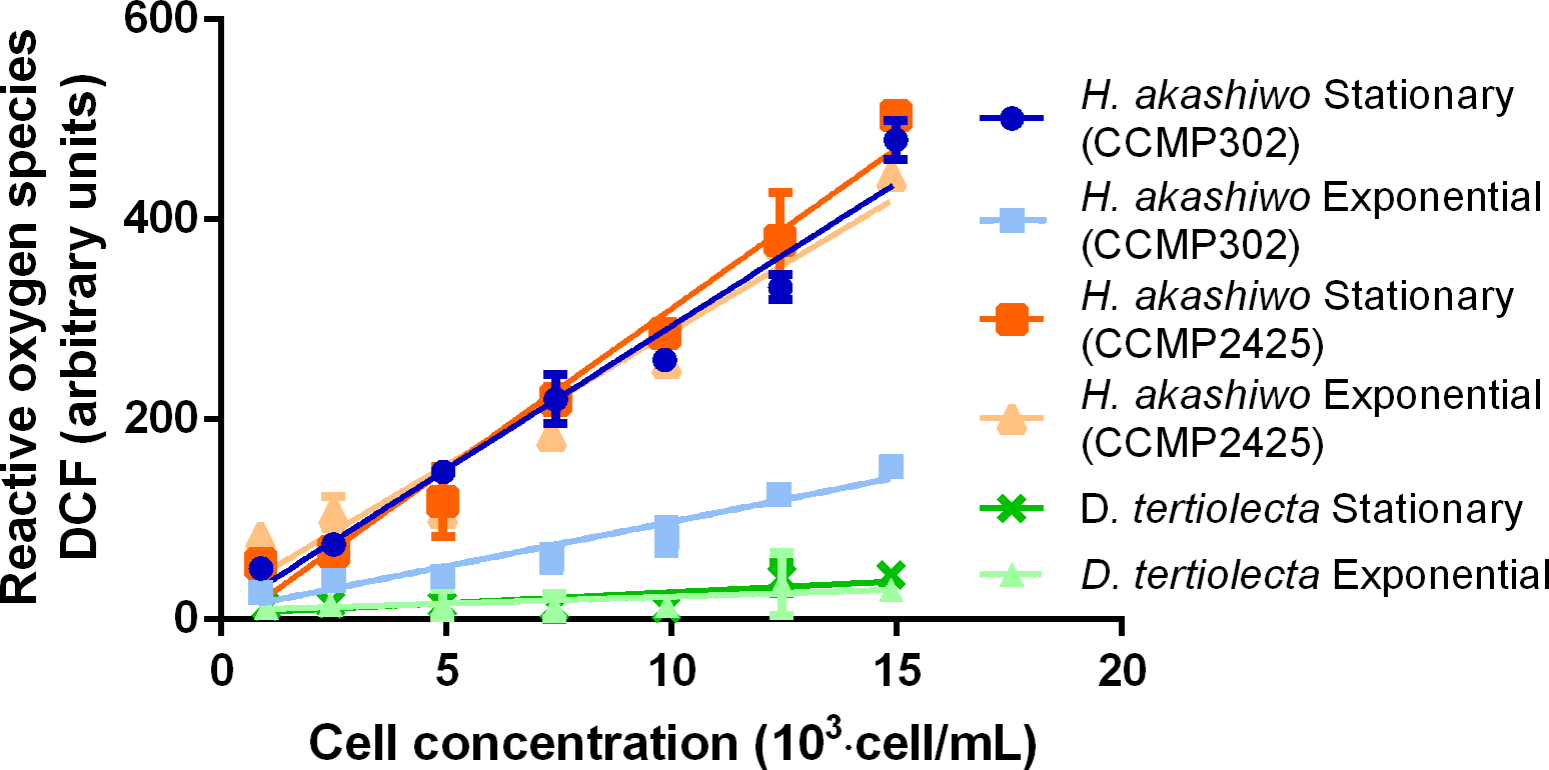
ROS-associated fluorescence emission of *H*. *akashiwo* (CCMP302 and CCMP2425) cells and *D*. *tertiolecta* cells in exponential and stationary phases, after incubation with H_2_DCFDA for different cell concentrations.

Cells of the *H*. *akashiwo* strains analyzed with the fluorescent probe DCF emitted fluorescence in both exponential and stationary growth phases, which was checked under a confocal microscope (S7 Fig). In general, fluorescence was distributed around the whole area of *H*. *akashiwo* cells and had a normal, healthy ovoid morphology in the exponential and stationary growth phases, showing a fluorescent signal, indicative of intracellular ROS generation. The fluorescence level measured in different growth phases from *Heterosigma* strain showed significant differences, with lower production during the exponential growth phase only for CCMP302 (Fig 5). The non-ichthyotoxic species, *D*. *tertiolecta*, showed much lower fluorescence levels compared to *H*. *akashiwo* strain and values were indistinguishable between growth phases (Fig 5).

## Discussion

In recent years, novel and sensitive *in vitro* approaches have been developed for the study of ichthyotoxicity mechanisms using fish cell lines [8,22,26]. The effect of ROS and other diffusible molecules can be assessed using this approach. In this study we determined the effect of *H*. *akashiwo* on Hsp70b transcription in fish CHSE-214 cells. These fish cells were selected due to their high cell adhesion and growth as a monolayer [26,32]. The expression of heat shock proteins (Hsps) is a widely used sub-lethal marker for environmental monitoring [33] that shows transcriptional changes induced by ROS and other stressors [25,31,34]. Our results indicated that the induced expression of Hsp70b by H_2_O_2_ was fast and clearly detected after 30 min. Furthermore, co-incubation assays between CHSE-214 cells and microalga were viable, which allowed for qPCR-based comparisons of effects of exposition to different microalga strains.

Thus, the effect of living *H*. *akashiwo* cells was detected through the increased expression of Hsp70b mRNA in Transwell co-culture with the fish cell line (Fig 1). Hydrogen peroxide (1μM) showed comparable expression to living *H*. *akashiwo* cells. Other assays were carried out to rule out the possibility that released toxins or bioactives could be contributing to the induced expression of Hsp70b. We did not observe any increase expression of Hsp70b mRNA for methanolic extract nor direct contact with living microalga (without Transwell). However, exponential supernatant of *H*. *akashiwo* also induced the overexpression of Hsp70b mRNA. In addition, SOD and catalase production (mRNA abundance) in fish cells exposed to *H*. *akashiwo* through the Transwell insert demonstrated that the increase expression of Hsp70b mRNA was due to ROS and not to biotoxins released after cell lysis. SOD activity was also higher when exponential cells assays were evaluated.

Fish cell line viability was not affected in our assays. Similarly, a previous study comparing several ichthyotoxic microalgae (including *H*. *akashiwo*) only found correlation among superoxide production, SOD activity and loss of viability for *C*. *marina* and *A*. *catenella* [8]. On the other hand, in our study, apoptotic cells were observed for direct contact and the total cell extract assays. There are previous evidence of apoptosis induction in Sf9 cells after exposure to *H*. *akashiwo* supernatants [19]. Although, in this case extracellular organic compounds were concentrated (x50). When direct-contact experiments were carried out, *H*. *akashiwo* cells rapidly stop swimming and settled to the bottom. This could be attributed to the change in osmolality that the presence of the Transwell membrane buffered. Then, this could be followed by passive (cell lysis) or active releasing of other molecules (including ROS, toxins, PUFAs). In this regard, Dorantes-Aranda et al. found loss of viability for gills cells exposed to an organic solvent extract of *H*. *akashiwo* but not for an aqueous one [8]. Thus, it was suggested that the toxic mechanism was due to hydrophobic compounds.

The proposed Transwell assay with the CHSE cells were used to compare two strains of *H*. *akashiwo* (Fig 3). As was shown, *H*. *akashiwo* CCMP2425 had a greater impact than the CCMP302 on the Hsp70 mRNA abundance. This could suggest that the former produced more ROS than the latter. Superoxide, hydrogen peroxide and hydroxyl radicals are produced by *H*. *akashiwo* and have been pointed out as the cause of its ichthyotoxicity [35]. In other studies, production of ROS is just one of the mechanisms, together with PUFAs and toxins, that has been proposed for explaining the harmful effect of *H*. *akashiwo* [35,36]. *Heterosigma akashiwo* has been found to produce a number of neurotoxins not completely identified [11,19]. ROS are extremely reactive and labile biomolecules whose determination is, therefore, troublesome [14]. Extracellular superoxide concentration has been generally used to assess and compare the ichthyotoxicity of different microalgae [8,37]. *H*. *akashiwo* releases lower quantities of superoxide than species such as *Chattonella* or *Karenia* but more than conventional microalgae [8]. In our study, extracellular superoxide production was greater for the ichthyotoxic species (both strains) than for the non-toxic microalga without differences among growth phases. To our best knowledge only exponential phase *Heterosigma* cells had been evaluated for superoxide production. With other raphidophytes there is conflicting evidence about whether ROS generation depends on the growth phase. On the one hand, Oda et al. (Oda et al., 1995) and Kim et al. (Kim, 2004) determined that superoxide production in *Chattonella* spp. was higher during the exponential growth phase and decreased during the stationary phase. On the other hand, Kim et al. (Kim, 2004) reported that H_2_O_2_ production by *Chattonella antiqua* was similar in both growth phases.

Our results showed a similar (CCMP2425) or lower (CCMP302) intracellular superoxide content for the ichthyotoxic species compared to non-toxic microalga (Fig 4A). Interestingly, superoxide levels through NBT assays were higher in the exponential growth phase for *H*. *akashiwo* strains. On the contrary, analysis in the non-toxic microalga *D*. *tertiolecta* showed elevated intracellular superoxide levels (higher than in *H*. *akashiwo* strain) in the stationary phase. As *D*. *tertiolecta* released lower levels of superoxide, this suggests a different role of superoxide among species (Fig 4B). Indeed, Twiner and Trick concluded that H_2_O_2_ released by *H*. *akashiwo* is not directly linked to photosynthesis [36]. According to our results, it is possible to conclude that there are differences in intracellular superoxide levels between growth phases, but no significant differences in extracellular superoxide release between growth phases. *H*. *akashiwo* strains clearly released more superoxide than the non-ichthyotoxic microalga. Consequently, it can be concluded that superoxide intracellular content and its release are not linked.

The fluorescent DCF probe, which accounts for intracellular ROS, offers some key advantages: for example, it can be oxidized by hydrogen peroxide and other ROS, such as peroxyl or hydroxyl radicals [38]. The approach used does not require cell lysis, thus allowing for *in vivo* detections [1]. In addition, the method only requires small sample volumes and short analysis times [38,39]. The obtained results with *H*. *akashiwo* and *D*. *tertiolecta* using the DCF probe for detecting intracellular ROS were not comparable to those from the intracellular NBT reduction analysis. The DCF assay showed higher ROS levels in the stationary phase of *H*. *akashiwo* (CCMP302) while NBT assay results indicated higher superoxide levels in the exponential phase (Fig 4). Strain CCMP2425 showed no differences between growth phases. Surprisingly, *D*. *tertiolecta* showed around 5 and 15 times lower level of ROS when compared to exponential CCMP302 cells or the rest of *H*. *akashiwo* cells evaluated, respectively. The DCF assay is generally used for ROS detection, but this probe can also be oxidized by reactive nitrogen species such as peroxynitrite and nitric oxide [38,40]. The observed differences might also be due to a differential penetration of DCF and NBT into subcellular compartments. Further experimentation is required to explain these observations. However, as was discussed for intracellular superoxide (NBT analysis), intracellular ROS is not a good indicative of extracellular ROS.

## Conclusions

The use of live microalgae and fish cell lines in Transwell co-cultures separated by a permeable membrane allowed for demonstrating the diffusion of soluble ROS inducing expression of SOD and the sub-lethal marker Hsp70b in a 30-min assay. The methodology avoided microalgae cell lysis and loss of viability in fish cell line allowing for co-culture up tp 6h. Biotoxins (evaluated as methanolic cell extract) did not induce expression of Hsp70b. Consequently, the methodology constitutes a valuable tool for assessing the ROS-mediated ichthyotoxic effect of microalgae. The evaluated *H*. *akashiwo* strains produced considerable intracellular ROS compared to non-toxic microalga, although it was not mostly superoxide. Superoxide release in ichthyotoxic strains was higher than in the non-ichthyotoxic microalga. It was demonstrated that living *H*. *akashiwo* cells in the Transwell assay caused Hsp70b induction which did not result in an acute loss of viability or apoptosis induction. On the other hand, direct contact of fish cells and *H*. *akashiwo* was followed by the appearance of apoptotic cells. Consequently, release of intracellular cell content (other than superoxide) can be pointed out as the main cause of ichthyotoxicity in *H*. *akashiwo*.

## Supporting information

S1 Fig Evolution of cell concentration for *Heterosigma akashiwo* (strains CCMP2425 and 302). (TIF)

S2 Fig Immunofluorescence images of CHSE-214 cells, (A) expressing vimentin (red) (B) TOPRO-3 (blue) for nuclear visualization. (C) Merge Vimentin (red) / Topro (blue), (D) Nomarsky (E) Phase contrast and counterstained with vimentin (red) (F) Phase contrast and TOPRO-3 (blue) for nuclear visualization. (F) Phasecontrast, stained with vimentin (red) and counterstained with Topro (blue).
(TIF)

S3 Fig Time evolution of mRNA levels of Hsp70 in CHSE-214 after exposure to H_2_O_2_. Legend. Results revealed increasing levels of Hsp70b mRNA expression after 1 h, and a plateau was reached between 2–6 h. A seven-fold increase in mRNA levels was finally observed (quantification relative to ß-actin expression).
(TIF)

S4 Fig CHSE-214 cell culture viability (based on MTT assay) after exposure to *H*. *akashiwo* in different assays. Microalga and fish cell culture media were used as negative control. H_2_O_2_ was used to prove that loss of viability was not due to oxidative stress.
(TIF)

S5 Fig Apoptosis in CHSE-214 cells following 6h of exposure to *H*. *akashiwo* stained with Acridine Orange and observed under fluorescence microscope. Negative Control (A-B). Direct-contact to stationary (C) and exponential (D) H. akashiwo. Transwell co-culture with stationary (E) and exponential (F) H. akashiwo. Arrows indicate the cell undergoing apoptosis and nuclear fragmentation.
(TIF)

S6 Fig Apoptosis in CHSE-214 cells following 6h of exposure to *H*. *akashiwo* stained with Hoechst and observed under fluorescence microscope. Negative Control (A-B). Direct-contact to stationary (C) and exponential (D) H. akashiwo. Transwell co-culture with stationary (E) and exponential (F) H. akashiwo. Arrows indicate the cell undergoing apoptosis and nuclear fragmentation.
(TIF)

S7 Fig Microscopy images of *H*. *akashiwo* (CCMP302) and *D*. *tertiolecta* (UTEX999). (A) and (B): H. akashiwo in stationary phase; (C) and (D): H. akashiwo in exponential phase. (E) and (F): D. tertiolecta in exponential phase. (A), (C) and (E): images obtained by using contrast phase microscopy. (B), (D) and (F): images obtained with laser confocal microscopy and H2DCFDA stain. (A—D scale bar: 50 μm; E and F scale bar: 100 μm).
(TIF)
