# Supplementary figures and images for: Study of the ichthyotoxic microalga Heterosigma akashiwo by transcriptional activation of sublethal marker Hsp70b in Transwell co-culture assays
Source: PLoS One. 2018 Aug 2;13(8):e0201438. doi: 10.1371/journal.pone.0201438 (PMC6072012; doi:10.1371/journal.pone.0201438)

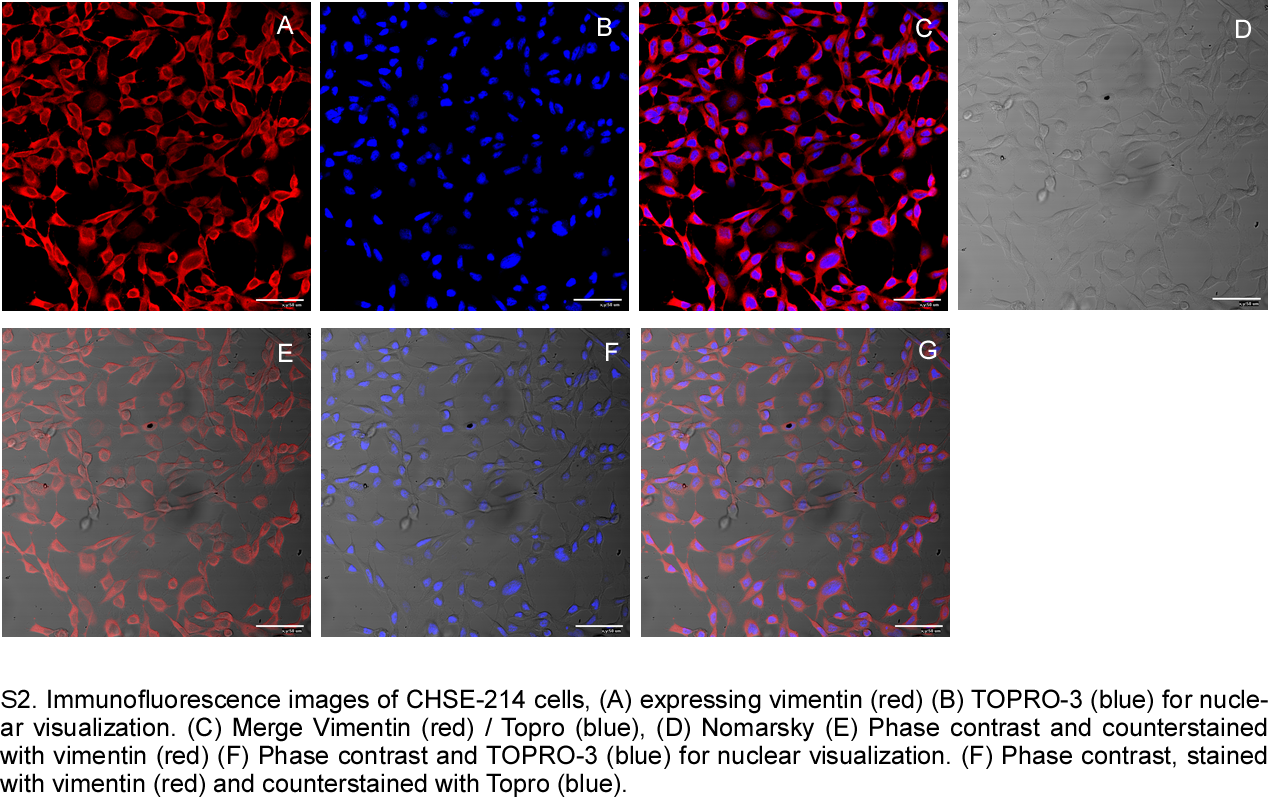

Supplement: S2 Fig — Immunofluorescence images of CHSE-214 cells, (A) expressing vimentin (red) (B) TOPRO-3 (blue) for nuclear visualization. (C) Merge Vimentin (red) / Topro (blue), (D) Nomarsky (E) Phase contrast and counterstained with vimentin (red) (F) Phase contrast and TOPRO-3 (blue) for nuclear visualization. (F) Phasecontrast, stained with vimentin (red) and counterstained with Topro (blue). (TIF) [file pone.0201438.s002.tif]

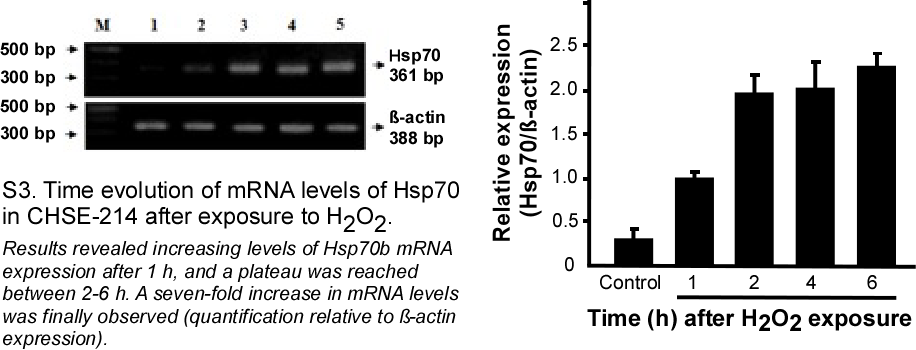

Supplement: S3 Fig — Legend. Results revealed increasing levels of Hsp70b mRNA expression after 1 h, and a plateau was reached between 2–6 h. A seven-fold increase in mRNA levels was finally observed (quantification relative to ß-actin expression). (TIF) [file pone.0201438.s003.tif]

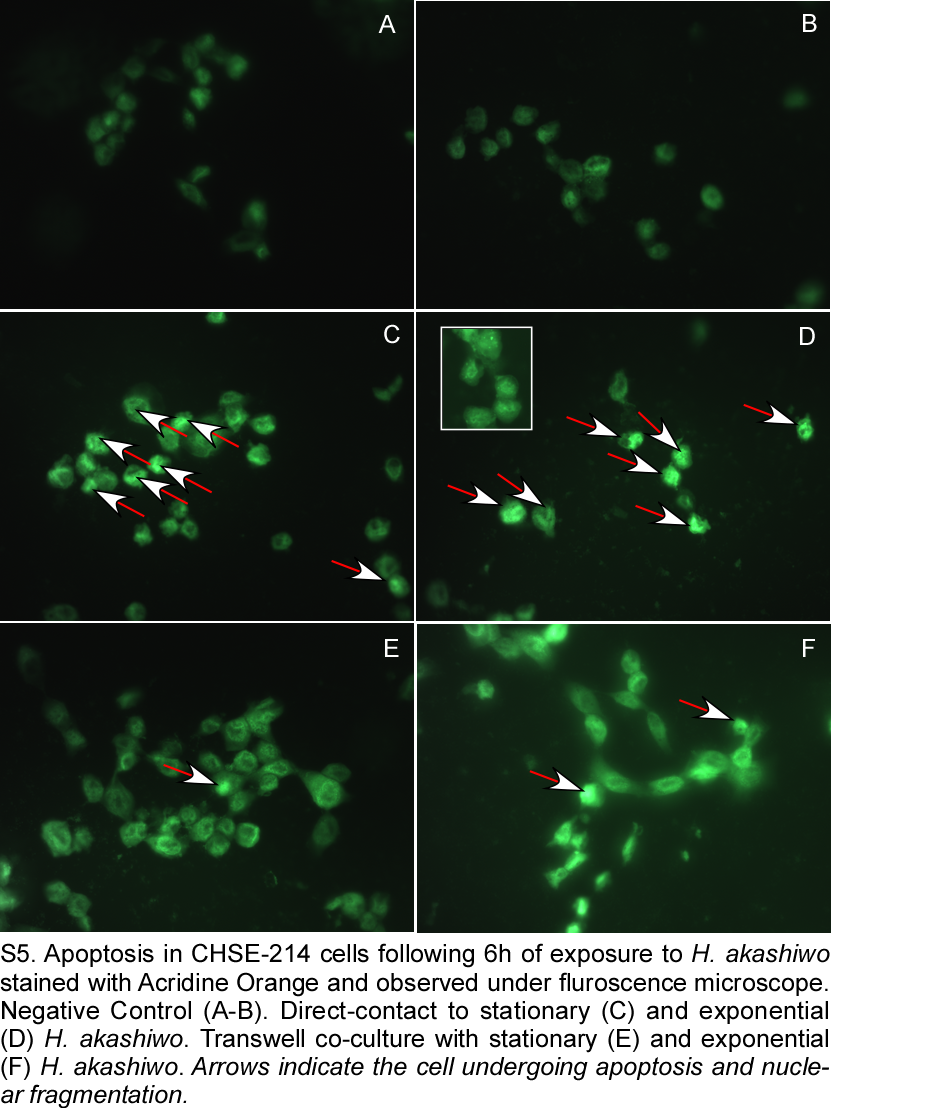

Supplement: S5 Fig — Negative Control (A-B). Direct-contact to stationary (C) and exponential (D) H. akashiwo. Transwell co-culture with stationary (E) and exponential (F) H. akashiwo. Arrows indicate the cell undergoing apoptosis and nuclear fragmentation. (TIF) [file pone.0201438.s005.tif]

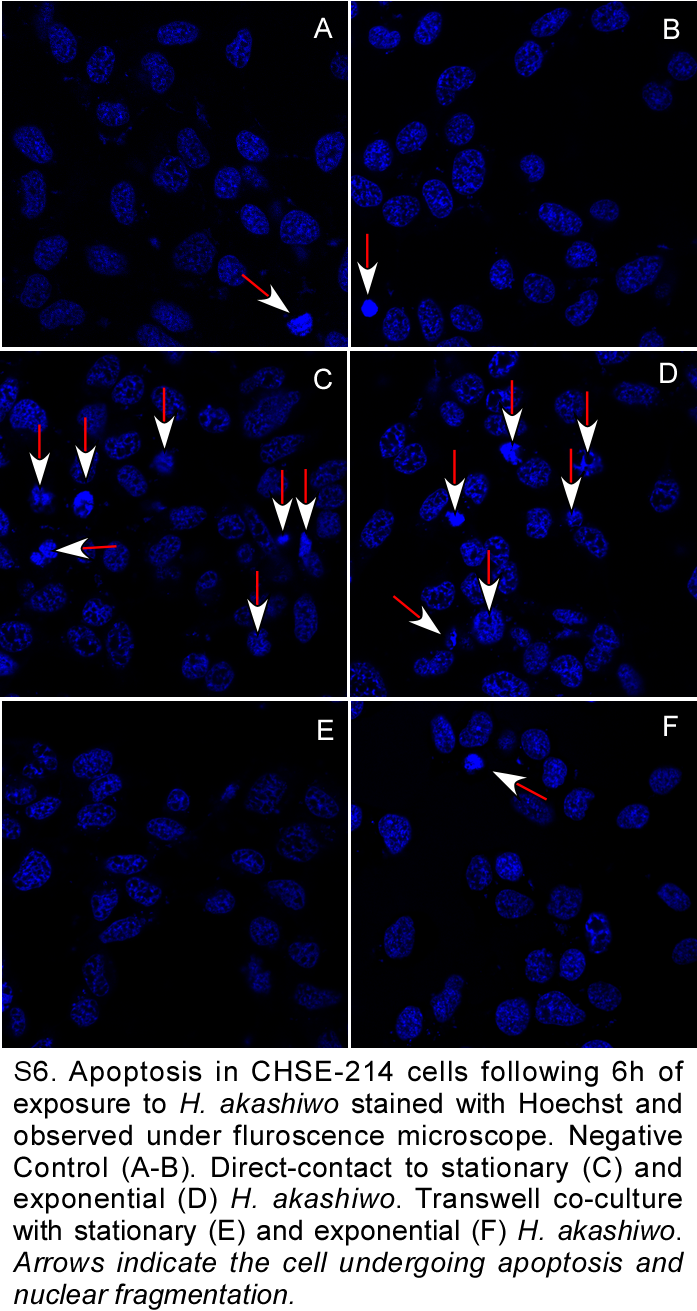

Supplement: S6 Fig — Negative Control (A-B). Direct-contact to stationary (C) and exponential (D) H. akashiwo. Transwell co-culture with stationary (E) and exponential (F) H. akashiwo. Arrows indicate the cell undergoing apoptosis and nuclear fragmentation. (TIF) [file pone.0201438.s006.tif]

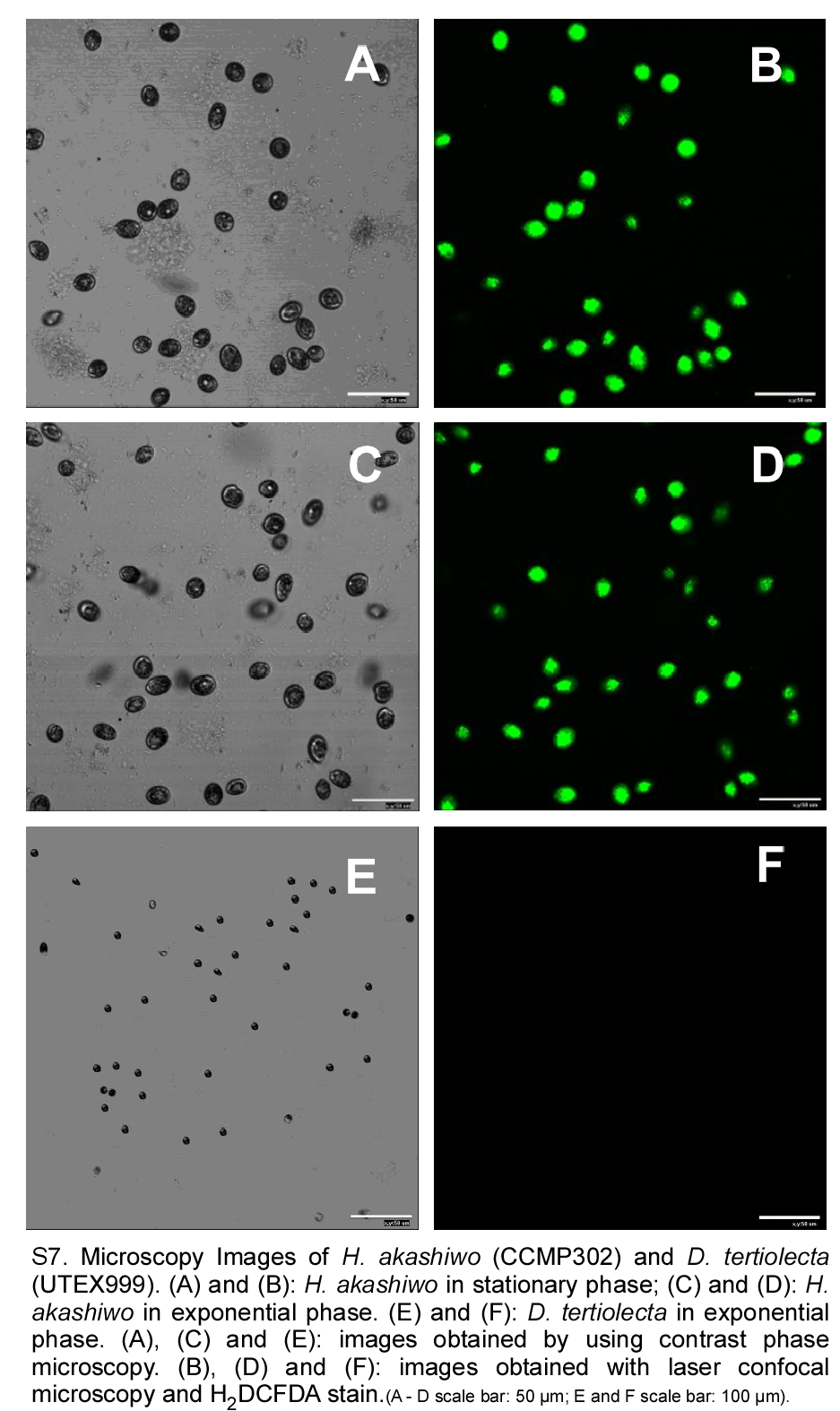

Supplement: S7 Fig — (A) and (B): H. akashiwo in stationary phase; (C) and (D): H. akashiwo in exponential phase. (E) and (F): D. tertiolecta in exponential phase. (A), (C) and (E): images obtained by using contrast phase microscopy. (B), (D) and (F): images obtained with laser confocal microscopy and H2DCFDA stain. (A—D scale bar: 50 μm; E and F scale bar: 100 μm). (TIF) [file pone.0201438.s007.tif]
